# The Critical Roles of the SUMO-Specific Protease SENP3 in Human Diseases and Clinical Implications

**DOI:** 10.3389/fphys.2020.558220

**Published:** 2020-10-30

**Authors:** Xiaojun Long, Biying Zhao, Wenbin Lu, Xu Chen, Xinyi Yang, Jifang Huang, Yuhong Zhang, Siming An, Yuanyuan Qin, Zhengcao Xing, Yajie Shen, Hongmei Wu, Yitao Qi

**Affiliations:** Key Laboratory of the Ministry of Education for Medicinal Resources and Natural Pharmaceutical Chemistry, National Engineering Laboratory for Resource Developing of Endangered Chinese Crude Drugs in Northwest of China, College of Life Sciences, Shaanxi Normal University, Xi’an, China

**Keywords:** SUMOylation, SENP3, cancer, neurological disease, cardiovascular disease

## Abstract

Post-translational modification by SUMO (small ubiquitin-like modifier) proteins has been shown to regulate a variety of functions of proteins, including protein stability, chromatin organization, transcription, DNA repair, subcellular localization, protein–protein interactions, and protein homeostasis. SENP (sentrin/SUMO-specific protease) regulates precursor processing and deconjugation of SUMO to control cellular mechanisms. SENP3, which is one of the SENP family members, deconjugates target proteins to alter protein modification. The effect of modification via SUMO and SENP3 is crucial to maintain the balance of SUMOylation and guarantee normal protein function and cellular activities. SENP3 acts as an oxidative stress-responsive molecule under physiological conditions. Under pathological conditions, if the SUMOylation process of proteins is affected by variations in SENP3 levels, it will cause a cellular reaction and ultimately lead to abnormal cellular activities and the occurrence and development of human diseases, including cardiovascular diseases, neurological diseases, and various cancers. In this review, we summarized the most recent advances concerning the critical roles of SENP3 in normal physiological and pathological conditions as well as the potential clinical implications in various diseases. Targeting SENP3 alone or in combination with current therapies might provide powerful targeted therapeutic strategies for the treatment of these diseases.

## Introduction

SUMO (small ubiquitin-like modifier) proteins were discovered more than two decades ago, and mammals express four SUMO paralogs, including SUMO1 with 101 amino acids, SUMO2 with 103 amino acids, and SUMO3 and SUMO4 with 95 amino acids (Kamitani et al., 1998; Johnson, 2004). SUMO modification (SUMOylation) is catalyzed by the SUMO-specific activating enzyme (E1), binding enzyme (E2) and ligating enzyme (E3), and they are entirely different from ubiquitin E1, E2, and E3 (Gong et al., 1997, 1999). As one of the post-translational modifications (PTMs), SUMO is catalytically conjugated to a large number of cellular protein substrates via SUMOylation, which has been discovered as a critical regulatory mechanism in many signaling pathways that alters the cellular localization, protein–protein interaction, and biological function of target proteins (Hay, 2005; Rytinki and Palvimo, 2009; Wang and Schwartz, 2010). SUMOylation regulates target proteins and thus plays a critical role in normal cellular activities by regulating many key biological processes, including DNA repair, gene transcription, and cell cycle progression (Ulrich, 2009; Yeh, 2009).

SUMOylation is a dynamically reversible process, and SUMOylated proteins are rapidly deconjugated via the proteins of the SENP (sentrin/SUMO-specific protease) family (Mukhopadhyay and Dasso, 2007; Yeh, 2009). SUMO precursors are processed by the corresponding SENPs, which possess endopeptidase activity, into their mature forms (Yeh et al., 2000). The SENP family members deconjugate SUMOylated protein substrates and function as isopeptidases by disrupting protein SUMOylation and maintaining cellular SUMOylation homeostasis (Yeh et al., 2000). The SENP family can be divided into three subfamilies, each with unique cellular localization, substrate specificity, and substrate specificity (Flotho and Melchior, 2013). The first subfamily includes SENP1 and SENP2, which have extensive substrate specificity (Cheng et al., 2007; Kang et al., 2010; Qi et al., 2014a,b; Wu et al., 2016; Chen et al., 2018). The second subfamily includes SENP3 and SENP5, which are both nucleolus proteins and specifically bind to SUMO2/3 (Gong and Yeh, 2006; Huang et al., 2009). The third subfamily includes SENP6 and SENP7, which are nucleoplasm proteins that display a clear proteolytic cleavage preference for SUMO2/3, and their catalytic domains have additional cyclic structures (Dou et al., 2010; Bawa-Khalfe et al., 2012). The SENP family plays an essential role in maintaining the balance between the SUMOylation and de-SUMOylation of target proteins and maintaining the normal physiological function in cells (Yeh et al., 2000; Hang and Dasso, 2002). The purpose of this review is to provide an overview of the most recent advances concerning the critical roles of SENP3 in normal physiological and pathological conditions and its potential clinical implications.

## SENP3 Regulates Intracellular Pathways and Cellular Activities

### General Features and Regulation of SENP3

Human SENP3 consists of 574 amino acids and is a 65 kDa protein (Kunz et al., 2018). Cys532 of SENP3 is a key nucleophile that attacks the isopeptide bond between the terminal Gly of SUMO and the Lys of substrates. SENP3 is abundant in the thymus and spleen and has little significance in the brain and heart tissue in humans (Yun et al., 2008; Kolli et al., 2010). SENP3 specifically plays an important role in SUMO2/3 precursor processing and deconjugation (Gong and Yeh, 2006). It was reported that SENP3 only has low isopeptidase activity against RanGAP1-SUMO1 and preferentially deconjugates SUMO2/3 from RanGAP1 (Kolli et al., 2010).

The SENP3 expression level is regulated by several PTMs. SENP3 is phosphorylated by the protein kinase CDK1 upon initiation of mitosis and is dephosphorylated by the protein phosphatase PP1α upon exit from mitosis (Wei et al., 2018). The mTOR kinase phosphorylates the N-terminal region of SENP3, leading to the translocation of SENP3 to the nucleolus and regulating the nucleolar targeting of SENP3 (Raman et al., 2014). Nucleophosmin (NPM) is a universally expressed nucleolar scaffold phosphoprotein, that is actively involved in the shuttling of ribosome components between the cytoplasm and the nucleus (Borer et al., 1989). NPM binds to SENP3, and it is critical for stable SENP3 accumulation in mammalian tissue culture cells (Yun et al., 2008). An earlier study showed that the tumor suppressor protein p19^*Arf*^ triggered the sequential phosphorylation and ubiquitination of SENP3, and SENP3 degradation occurred through the ubiquitin-proteasome pathway (Kuo et al., 2008). The ability of p19^*Arf*^ to enhance SENP3 turnover depends on the presence of NPM.

The molecular chaperone heat shock protein 90 (Hsp90) refolds misfolded proteins and delivers damaged proteins to the ubiquitin-proteasome degradation machinery (Wegele et al., 2004). The chaperone/ubiquitin ligase carboxyl terminus of Hsc70-interacting protein (CHIP) binds to the Hsp90 protein and serves as a ubiquitin E3 ligase (Zhang et al., 2005). SENP3 is mostly located in the nucleolus of the cell, and the expression of SENP3 protein is increased by mild oxidative stress via interaction with CHIP and Hsp90 (Yan et al., 2010). When cells are treated with low doses of H_2_O_2_, the thiols of cysteines 243 and 274 in SENP3 undergo oxidative modification, which recruits Hsp90. Hsp90 and SENP3 association protects SENP3 from CHIP-mediated ubiquitination and subsequent degradation, enhancing the stability of the SENP3 protein (Yan et al., 2010). Mild oxidative stress enhances the stability of SENP3, displaces SENP3 from the nucleolus to the nucleus and modifies the function of its SUMOylated substrates (Wang et al., 2012b). SENP3 is phosphorylated and ubiquitinated, and other PTMs may also be involved in the regulation of SENP3 function.

### Roles of SENP3 in Ribosome Remodeling

Proline, glutamic acid, and leucine-rich protein 1 (PELP1) is a large multidomain protein that has been shown to regulate transcriptional activity as a cotranscription factor involved in chromosomal remodeling and cytoplasmic signal transduction (Girard et al., 2014). PELP1 is SUMOylated, and SUMOylated PELP1 recruits MDN1 to pre-60S particles. On the other hand, SENP3 deconjugates SUMOylated PELP1, which is required for the release of both factors during mammalian preribosome remodeling (Raman et al., 2016). Increasing evidence has shown that SENP3 participates in ribosome biosynthesis and maturation of ribosomal RNA (Haindl et al., 2008; Finkbeiner et al., 2011; Raman et al., 2014), indicating the important roles of SENP3 in ribosome remodeling.

### Roles of SENP3 in Autophagy

Autophagy is a highly conserved catabolic process induced after cellular stress to prevent cell damage, promote survival, and respond to various forms of cytotoxic damages (Dikic and Elazar, 2018). Beclin1, the mammalian homolog of the yeast autophagy-related gene ATG6, is widely expressed in normal human tissue and is an essential gene for mammalian autophagy. In the process of mammalian cell autophagy, Beclin1, Vps34, and ATG14L form a core protein complex. It was recently reported that SENP3 deSUMOylated Beclin1 to weaken the binding of Beclin1 with complex components and suppressed Vps34 activity, ultimately inhibiting the basal and starvation-induced autophagy in the liver (Liu et al., 2019). The critical role of SENP3 in autophagy may also be observed in other tissues, where it targets Beclin1 or different substrates.

### Roles of SENP3 in Mitosis and Cell Cycle Progression

Mitosis is the basis of eukaryotic cell proliferation, which ensures the consistency of the genetic composition of cells and tissues (Crncec and Hochegger, 2019). SUMO participates in cell mitosis by modifying Borealin in the chromosome passenger complex. SENP3 binds specifically to Borealin and removes its SUMO modification, which in turn affects the process of mitosis (Klein et al., 2009). SENP3 phosphorylation plays an important role in the regulation of the SUMOylation of chromosome-associated proteins and chromosome stability during mitosis (Huang et al., 2015; Wei et al., 2018). The expression of SENP3 was induced by low-dose H_2_O_2_ exposure, and the degree of PML SUMOylation was decreased, and the cell cycle was accelerated (Han et al., 2010). Mdm2 mediates p53 ubiquitination and degradation and regulates cell cycle progression (Honda et al., 1997). It was reported that SENP3 interacts with and deSUMOylates both mdm2 and p53, attenuating the effect of Mdm2 on p53 stability (Nishida and Yamada, 2011) and indicating that SENP3 may serve as a novel regulator of the Mdm2-p53 pathway during cell cycle progression. p19^*Arf*^ triggers the sequential phosphorylation and ubiquitination of SENP3, and the ubiquitin-proteasome mediated degradation of SENP3 induces cell cycle arrest (Kuo et al., 2008). These studies showed the critical roles of SENP3 in cell mitosis and cell cycle progression.

### Roles of SENP3 in Cell Survival Pathways

**FIGURE 1:**
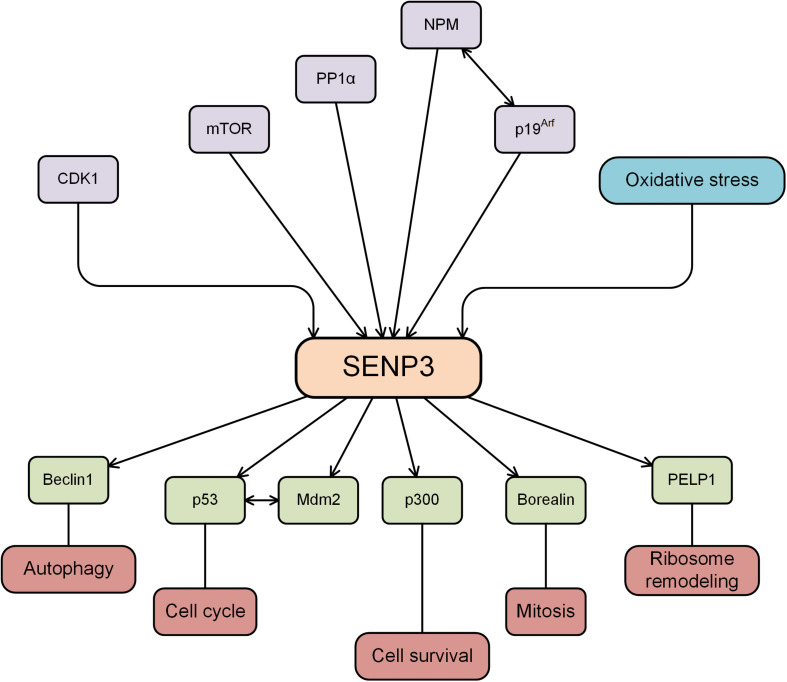
SENP3 is regulated by several proteins, while SENP3 deSUMOylates substrates and plays an important role in various cellular activities.

It was reported that there was a decreased level of SENP3 in PC12 cells treated with the toxic heavy metal cadmium. SENP3 degradation increased hyper-SUMOylation, which is protective against caspase-3-dependent cytotoxicity induced by cadmium (Luo et al., 2017). Biological oxidation is the most basic biochemical reaction in cells. Oxidative stress is also a critical mechanism required for the human body to protect itself and maintain cellular homeostasis, and it is one mechanism by which cells respond by activating pathways associated with cell survival or cell death (Battistelli et al., 2016). Hypoxia-inducible factor-1α (HIF-1α) is a well-known transcription factor that broadly controls gene expression in response to hypoxia. As an oxidative stress-sensitive molecule, SENP3 alters the transcriptional activity of HIF-1α by reversing the SUMOylation of the transcriptional coactivator p300 under oxidative stress (Huang et al., 2009; Wang et al., 2012a). SENP3 plays critical roles in the regulation of various cellular activities, and the variation of the SENP3 expression level is closely related to various diseases, including the occurrence and development of tumors, cardiovascular diseases, and neurological diseases (Figure 1).

## SENP3 and Cancer Occurrence, Development, and Progression

### Gastric Cancer

Gastric cancer is one of the most common malignancies of the digestive system in the world, and it has the second-highest incidence and mortality rate of all cancers (Song et al., 2017). Sp1 (specificity protein 1) is a cancer-associated transcription factor, and the level of Sp1 was significantly increased in parallel to the SENP3 level in gastric cancer cell lines, nude mice, and specimens derived from gastric cancer patients. RNF4 mediates Sp1 ubiquitination and proteasome degradation, whereas SENP3 can reduce Sp1 degradation via the deconjugation of SUMO2/3 from Sp1 and prevent RNF4 recognition and binding to Sp1, leading to the upregulation of Sp1 protein level. These studies indicated the important role of the enrichment of Sp1 protein, which is regulated by SENP3 and RNF4, in gastric cancer (Wang et al., 2016). Mesenchyme forkhead box C2 (FOXC2) is a transcription factor that has been identified to be induced by the epithelial–mesenchymal transition (EMT) (Taube et al., 2010). SENP3 promotes EMT in gastric cancer cells by regulating the EMT inducer FOXC2. In addition, the study found that mesenchymal marker gene expression and cell migration were enhanced in gastric cancer cells highly expressing SENP3. The correlation between SENP3 and gastric cancer metastasis was proven by the nude mouse model and patient specimens. The SENP3 level is increased in gastric cancer cells, and SENP3 deSUMOylates FOXC2, potentiates its transcriptional activity, induces the epithelial cell to interstitial cell changes, and promotes tumor invasion and metastasis (Ren et al., 2014). Taken together, the evidence shows that the expression level of SENP3 is increased in gastric cancer, and the increase in SENP3 increases deSUMOylation and enhances the tumorigenic activity of target proteins (Table 1).

**TABLE 1:** Biophysical and biological effects of SENP3-mediated deSUMOylation of protein substrates in human diseases.

**Human disease**	**Protein substrate**	**Biophysical function**	**Biophysical and biological effects of SENP3**
AMI	-	-	Brain ischemia dramatically alter SENP3 level, and this may be applied to promote cell survival after ischemia-reperfusion in the heart (Rawlings et al., 2019)
AML	NPM1	Molecular chaperone with multiple functions	SENP3 mediates deSUMOylation of NPM1, involving in the resistance of AML cells to therapy (Xu et al., 2019)
Cognitive dysfunction	Drp1	Required for mitochondrial division in mammalian cells	SENP3 mediates deSUMOylation of Drp1, acting as a critical regulator in sevoflurane induced cognitive dysfunction (Guo et al., 2017; Zheng et al., 2020)
	Rac1	A member of the Rho family of small GTPases	SENP3 deSUMOylates and activate Rac1, and reduces hippocampal synaptic plasticity and impairs cognitive function (Yang et al., 2019)
Gastric cancer	FOXC2	EMT induced transcription factor	SENP3 potentiates the transcriptional activity of FOXC2 through deSUMOylation, inducing mesenchymal gene expression in metastasis of gastric cancer (Ren et al., 2014)
	Sp1	Cancer-associated transcription factor	SENP3 inhibits RNF4-mediated Sp1 ubiquitination and degradation, leading to up-regulation of Sp1 level (Wang et al., 2016)
Glioma	Sp3	Ubiquitously expressed transcription factor, participates in cell proliferation, apoptosis, and differentiation	The deSUMOylation of Sp3 results in an up-regulation of MAOB level, and an increase in cellular peroxide levels (Sharpe and Baskin, 2016).
HNC	STAT3	Phosphorylated STAT3 regulates genes involved in cell proliferation, apoptosis, differentiation and survival	SENP3 enhances the basal and NNK or IL-6 induced phosphorylation of STAT3 in HNC cells (Zhou et al., 2016)
Inflammation	BACH2	A well-known transcriptional repressor regulating both B and T lymphocyte differentiation and maturation, and is essential for the maintenance of immune tolerance and homeostasis mediated by Treg cells	SENP3 plays a critical role in the maintenance of regulatory T cell stability and function via BACH2 deSUMOylation, and regulates ROS induced immune tolerance (Yu et al., 2018)
	IQGAP2	One Ras GTPase activating like protein, regulates cell cytoskeleton, cell adhesion, and apoptosis	IQGAP2 is SUMOylated, whereas SENP3 mediated deSUMOylation of IQGAP2, and the host defense restores host protein synthesis and suppressed the expression of the HBV gene (Xi et al., 2019)
	MKK7	Promotes the phosphorylation of JNK, and then activate AP-1 to enter the nucleus and initiate the inflammatory response	The decrease of SENP3 increases the SUMOylation of MKK7, reduces the interaction between MKK7 and JNK and inflammatory response, and improves the survival rate of the model mice (Lao et al., 2018)
	NLRP3	Component of the inflammasome	SENP3 deSUMOylates NLRP3 to inhibit ASC recruitment, speck formation, and NLRP3 inflammasome activation (Shao et al., 2020)
Ischemia	Drp1	Required for mitochondrial division in mammalian cells	SENP3 deficiency increases Drp1 SUMOylation, suppresses cytochrome c release cell death (Guo et al., 2013)
Myopathy and cachexia	SETD7	Histone lysine methyltransferase	SENP3 deSUMOylation of SETD7 is impaired in cachexia, leading to the dramatic loss of sarcomeric protein (Nayak et al., 2019)
NAFLD	A2M, APOE, TNFRSF11B	Regulates downstream genes regulating lipid metabolism	Hepatic SENP3 is up-regulated in NAFLD patients (Liu Y. et al., 2016)
NVU dysfunction	-	-	SENP3 is increased in the hippocampal CA1 following CCH, and chronic treatment with FAAH inhibitor decreases SENP3 and ameliorates CCH-induced NVU impairment (Wang et al., 2017)
OSCC	-	-	SENP3 is highly expressed and plays a critical role in carcinogenesis and differentiation of OSCC under oxidative stress (Sun et al., 2013)
Osteoporosis	IRF8, RbBP5	Prevent estrogen-deficient osteoporosis	SENP3 deSUMOylates IRF8 and RbBP5, and suppresses osteoclast differentiation (Zhang et al., 2020)
Ovarian cancer	E-cadherin, FOXC2, N-cadherin, p21, PCNA	Related to cell proliferation, metastasis and invasion	SENP3 promotes cell proliferation, metastasis, and tumorigenesis, and is a redox-sensitive molecule mediating the EMT (Cheng et al., 2017)
Preeclampsia	FIH1	Regulates the transcriptional activity of HIF1A	SUMOylation of FIH1 directly impacted the transcriptional activity of HIF1, and SENP3 reversed the effect during placental development (Sallais et al., 2017)
	HIF1A	Plays an integral role in the cellular response to low oxygen concentrations, or hypoxia	SENP3 reduces the degradation of HIF1A and maintains the stability of HIF1A during placental development (Bhattacharjee et al., 2016)
SAH	-	-	SENP3 induces neuronal apoptosis and brain damage after SAH (Yu et al., 2015)
SCI	-	-	SENP3 plays an important role in neuronal apoptosis, and participates in the physiological and pathological processes of SCI (Wei et al., 2012; Luo et al., 2017)

### Glioma

Glioma is one of the most aggressive malignancies occurring in the nervous system with high incidence and mortality worldwide, and the current clinical treatment for glioblastoma is palliative but not curative (Preusser et al., 2011). The ubiquitously expressed transcription factor Sp3 (specificity protein 3) participates in the expression of genes involved in cell differentiation, proliferation and apoptosis (Cavanaugh and Dimario, 2017). Sp3 is functionally regulated by SUMOylation, and SENP3 mediates the deSUMOylation of Sp3 in glioma (Stielow et al., 2010). Monoamine oxidase B (MAOB) levels are correlated with the glioma tumor grade. It was previously shown that SENP3 regulates the transcriptional activity of HIF1α by reversing the SUMOylation of p300, and elevation of peroxide increases the levels of the active HIF1α/p300 complex (Huang et al., 2009). The study indicated that the expression of MAOB is correlated with HIF1α expression levels. A previous study showed that SENP3 mediated the deSUMOylation of Sp3, resulting in an increased level of MAOB and further leading to increased cellular peroxide levels (Sharpe and Baskin, 2016). These results indicated the important role of SENP3-mediated deSUMOylation of Sp3 in glioma.

### Head and Neck Cancer

Head and neck cancer (HNC) or head and neck squamous cell carcinoma, which occurs in the upper aerodigestive tract, is the most common epithelial malignancy (Song and Grandis, 2000). The activation of Nrf2 in laryngeal carcinoma is stimulated by cisplatin-induced ROS (reactive oxygen species) production through the deSUMOylation activity of SENP3 (Zhou et al., 2019). STAT3 (signal transducer and activator of transcription 3) is hyperphosphorylated and activated early during HNC carcinogenesis (Nagpal et al., 2002). Phosphorylation of STAT3 at tyrosine 705 is necessary for its dimerization and nuclear translocation, and it is considered a typical marker for cytokine or growth factor-induced STAT3 activation. It was demonstrated that SENP3 protein levels are positively correlated with smoking and STAT3 Y705 phosphorylation levels in laryngeal carcinoma. In addition, it was demonstrated that tobacco extract induced phosphorylation of Y705 in STAT3 and an increase in SENP3 in HNC cell lines, and these phenomena were dependent on the simultaneous increase in ROS. SENP3 deconjugates the SUMO2/3 modification of STAT3 and then enhances STAT3 phosphorylation. SUMOylation is a previously undescribed PTM of STAT3, and SENP3 acts as an essential regulator of STAT3 activation induced by tobacco or cytokines (Zhou et al., 2016). These findings provide novel insights into the role of the SENP3-mediated hyperphosphorylation of STAT3 in the development of HNC.

### Oral Squamous Cell Carcinoma

Oral squamous cell carcinoma (OSCC) is one of the most common types of throat malignant tumors in Southeast Asia, and there are more than 300000 new patients diagnosed annually worldwide (Kademani, 2007). A previous study indicated that the expression level of SENP3 was significantly increased and is associated with pathological characteristics in OSCC tissues compared to normal mucosa tissue adjacent to the tumor. It was concluded that SENP3 is highly expressed and may play a critical role in the carcinogenesis and differentiation of OSCC under oxidative stress (Sun et al., 2013). However, the specific role of SENP3 and the target proteins regulated by SENP3 in OSCC need further study.

### Ovarian Cancer

Ovarian cancer is one of the most common forms of gynecologic malignancies and causes more mortality than any other female reproductive cancer (Siegel et al., 2020). SENP3 expression is increased in ovarian cancer compared with that in normal tissues. When SENP3 was knocked down, the proliferation, metastasis, and invasion of ovarian cancer cells were greatly weakened, and the expression levels of proliferating cell nuclear antigen (PCNA), FOXC2, and neuronal cadherin (N-cadherin) were decreased. In contrast, the expression of p21 and epithelial-cadherin (E-cadherin) was increased. It was verified that SENP3 promoted cell proliferation, metastasis, and tumorigenesis in ovarian cancers as a redox-sensitive molecule mediating EMT in ovarian cancer cells. Therefore, SENP3 might play a critical role in the progression of epithelial ovarian cancer, and SENP3 could serve as a potential biomarker for the prognosis of ovarian cancer. Ultimately, the regulation of SENP3 may provide a promising therapeutic target for ovarian cancer (Cheng et al., 2017).

## Neurological Diseases

### Cognitive Function

CC2D1A (coiled-coil and C2 domain-containing 1A) was the first identified member of the Cc2d1 gene family and contained a Drosophila melanogaster 14 (DM14) domain, protein kinase C conserved region 2 (C2) domain, and predicted helix-loop-helix DNA-binding domain (Ou et al., 2003). Rac1 is a member of the Rho family of small GTPases, and a previous study showed that there were enhanced Rac1 activity in synaptic plasticity and cognitive deficits in Cc2d1a conditional knockout (cKO) mice. The enhancement of Rac1 activity was mediated by the increase in Rac1 SUMOylation via the reduction in SENP1 and SENP3 expression (Yang et al., 2019). Both SENP1 and SENP3 are essential for the maintenance of hippocampal CA1 LTP and long-term objective location memory (OLM) performance.

Sevoflurane is one of the most common medicines used as an inhaled anesthetic for clinical surgery (Liang et al., 2017). Previous studies have shown that the use of sevoflurane in older adults can increase the incidence of POCD (post-operative cognitive dysfunction) (Geng et al., 2017). Drp1 (dynamin-related protein) is essential for mitochondrial division in mammalian cells, and Drp1 SUMOylation is linked to the Drp1 activity cycle (Figueroa-Romero et al., 2009). Mechanistically, these studies showed that exposure to sevoflurane increased the expression level of SENP3 and Drp1 deSUMOylation in the hippocampal area and ultimately resulted in cognitive deficiency. SENP3-mediated deconjugation of SUMOylated Drp1 serves as a critical regulator in sevoflurane-induced cognitive dysfunction (Zheng et al., 2020). SENP3 plays a critical role in the regulation of cognitive function through deSUMOylation of Rac1 and Drp1 and other potential targets.

### Ischemia

Brain ischemia is one of the major causes of disability and death, and it occurs when the blood supply to the brain is interrupted by occlusion or trauma after heart failure or stroke (Guo et al., 2013). In response to ischemia, Drp1 in the mitochondrial outer membrane (MOM) plays a critical role in initiating cell death, and prevention of Drp1-mediated mitochondrial fission is cytoprotective (Hall et al., 2014; Guo et al., 2017). SENP3 regulates Drp1 deSUMOylation, enhances Mff (mitochondrial fission factor)-mediated mitochondrial recruitment, and ultimately promotes cell death (Guo et al., 2017). SENP3 was degraded in an *in vitro* model of ischemia during oxygen/glucose deprivation (OGD). The depletion of SENP3 promotes Drp1 SUMOylation, and the hyper-SUMOylation of Drp1 suppresses Drp1-mediated cytochrome c release and caspase-mediated cell death. It was concluded that the dynamic changes in SENP3 stability and SENP3-regulated Drp1 deSUMOylation are critical determinants of cell fate during ischemia (Guo et al., 2013).

### Neurovascular Unit Dysfunction

The concept of the neurovascular unit (NVU) comprises neurons, glial cells, vascular smooth muscle cells, endothelial cells, and ECM (extracellular matrix) (Sweeney et al., 2016). Structural brain connectivity, synaptic activity, and information processing require highly coordinated signal transduction among different cell types and non-cell elements within the functional NVU and intact BBB (blood–brain barrier) (Zhao et al., 2015). NVU disruption has been broadly observed in vascular dementia, Alzheimer’s disease, and ischemic cerebrovascular diseases that are associated with CCH (chronic cerebral hypoperfusion) (Shang et al., 2016). The expression level of SENP3 was increased in the hippocampal CA1 area following CCH. Chronic treatment with the fatty acid amide hydrolase (FAAH) inhibitor URB597 ameliorates CCH-induced NVU impairment by reducing SENP3 levels (Wang et al., 2017). These results indicated that SENP3 might play an important role in NVU; however, the molecular mechanism of this process needs further investigation.

### Subarachnoid Hemorrhage (SAH)

Subarachnoid hemorrhage (SAH) is a life-threatening disease with high lethality and disability rates (Hasegawa et al., 2011). Researchers found that the expression of SENP3 in neurons was increased after SAH, while the expression of SENP3 in astrocytes and microglia did not change. Moreover, suppression of SENP3 promotes the inhibition of apoptosis in experimental SAH in rat models (Yang et al., 2015). In addition, caspase-3 also has a positive correlation with SENP3 (Yu et al., 2015). Therefore, SENP3 may induce neuronal apoptosis and brain damage after SAH, whereas the particular roles of SENP3 and the potential target proteins need further study.

### Spinal Cord Injury (SCI)

Spinal cord injury (SCI) is a severely disabling disease and is extremely costly with long-term consequences (Johnson et al., 1997). The expression of SENP3 in an acute SCI model was increased and located in neuronal cells, which was involved in the apoptosis of neurons. There was significant upregulation of SENP3 with activation of caspase-3 following stimulation. In addition, the study found that the expression level of SENP3 in white matter and gray matter was very low under normal conditions. However, in the acute SCI model of adult mice, the expression of SENP3 3 days after SCI continued to rise until the peak was reached, and then it decreased. Moreover, the study also found that the expression of caspase-3 increased significantly after SCI. Researchers applied H_2_O_2_ to induce the apoptosis of neurons *in vitro*, and the study found that both SENP3 and active caspase-3 were significantly upregulated in tandem, indicating that SENP3 may play a role in neuronal apoptosis and participate in the physiological and pathological processes of SCI (Wei et al., 2012). However, whether SENP3 has a direct effect on neuronal apoptosis requires further investigation.

## Acute Myocardial Infarction

Acute myocardial infarction (AMI) occurs after a reduction in myocardial perfusion and is a leading cause of mortality (Pu et al., 2017). In the H9C2 cardiomyocyte cell line, the expression level of SENP3 was increased after hypoxia-reoxygenation, whereas knockdown of SENP3 promoted apoptosis after hypoxia-reoxygenation. Further study indicated that SENP3 protected H9C2 cells against hypoxia-reoxygenation by enhancing the JAK2/STAT3 pathway, which was followed by increased expression of Bcl-2/Bax and the inactivation of caspase-3-mediated apoptosis (Zhang et al., 2018). Another study used the Langendorff perfusion model to detect the level of SENP3 in the whole heart of rats. There was a 50% decrease in SENP3 levels in the cytosolic fraction of the heart after preconditioning, a 90% loss after ischemia and an 80% loss after ischemia-reperfusion. Furthermore, knockdown of SENP3 in H9C2 cells led to an increased cell death rate upon reperfusion, suggesting that SENP3 plays a cardioprotective role during ischemia-reperfusion (Rawlings et al., 2019). These studies similarly showed that the increase in SENP3 played a cardioprotective role in whole heart and H9C2 cells during ischemia-reperfusion. However, another study reported that the expression level of SENP3 was upregulated in the heart tissue of mice after ischemia-reperfusion and that knockdown of SENP3 significantly reduced the infarct size and improved cardiac function in mice through the endoplasmic reticulum (ER) stress and mitochondria-mediated apoptosis pathways (Gao et al., 2018). The study indicated that SENP3 contributes to myocardial ischemia-reperfusion injury in mice, which is in contrast to the results of previous studies. One reason for this is that the later study was performed in mice, whereas previous studies used hearts isolated from rats and H9C2 cells. Further studies are needed to clarify the critical role of SENP3 during ischemia-reperfusion. Since SENP3 knockout is embryonic lethal in mice (Lao et al., 2018), SENP3 conditional knockout in cardiomyocytes may be an appropriate model. These results indicated that cardiac ischemia dramatically alters the SENP3 level, and the regulation of the SENP3 level may be applied to promote the survival of cardiomyocytes after ischemia-reperfusion in the heart.

## SENP3 Mediates the Resistance of Acute Myeloid Leukemia Cells to Medical Therapy

Acute myeloid leukemia (AML) is a serious hematological malignant disorder (Zheng and Studzinski, 2017). The promyelocytic leukemia (PML) protein regulates cell apoptosis, cell cycle, and DNA damage responses. In mammalian cells, PML is mainly enriched in a discrete nuclear substructure called the PML nuclear body (NB), which recruits proteins and promotes protein modifications (Hsu and Kao, 2018). SENP3 regulates the deSUMOylation of PML and alters the ability of the PML protein to form NBs in the nucleus, and PML deSUMOylation is correlated with increased cell proliferation under mild oxidative stress (Han et al., 2010). A previous study showed that NPM1 gene mutations are the most common genetic lesions in AML patients (Martelli et al., 2015). NPM1 is modified by SUMO, and SENP3 reverses its SUMOylation status. NPM1 SUMOylation is essential for the recruitment of DNA repair proteins at the early stage of DDR (DNA damage response), and SUMOylated NPM1 affects the assembly of the BRCA1 complex, which plays a critical role in the DNA damage repair mechanism (Manke et al., 2003). hCINAP (human coilin-interacting nuclear ATPase protein) is a partner protein of NPM1 and participates in the repair of DNA DSB (double-stranded break), and it was identified that the expression of hCINAP is correlated with AML prognosis. Knockdown of hCINAP also sensitized a PDX (patient-derived xenograft) mouse model to chemotherapy, and SENP3-mediated deSUMOylation of NPM1 is involved in the resistance of AML cells to therapy (Xu et al., 2019).

## SENP3 Enhances the Inflammatory Response Signaling Pathway

The innate inflammatory response in the human body is precisely controlled to maintain the balance between tissue integrity and pathogen clearance (Liu J. et al., 2016). SENP3 plays an important role in the production of lipopolysaccharide (LPS)-induced cytokines. MKK7 (mitogen-activated kinase kinase 7) specifically regulates the phosphorylation of JNK and then activates AP-1, which allows it to enter the nucleus and initiate the inflammatory response (Park et al., 2019). SENP3 effectively promotes MKK7 deSUMOylation, increases the interaction of JNK and MKK7, promotes the phosphorylation of JNK, and then activates AP-1. AP-1 enters the nucleus as an inflammatory factor to initiate an inflammatory response. The decrease in SENP3 increases the SUMOylation of MKK7, reduces the interaction between MKK7 and JNK and the inflammatory response, and improves the survival rate of mice (Lao et al., 2018). BACH2 (BTB domain and CNC homolog 2) is a well-known transcriptional repressor regulating both B and T lymphocyte differentiation and maturation and is essential for the maintenance of immune tolerance and homeostasis mediated by Treg cells (Roychoudhuri et al., 2013). SENP3 also plays a critical role in the maintenance of regulatory T cell stability and function via BACH2 deSUMOylation and regulates ROS-induced immune tolerance (Yu et al., 2018). IQGAP2 (IQ motif-containing GTPase-activating protein 2), a Ras GTPase-activating-like protein, regulates the cell cytoskeleton, cell adhesion, and apoptosis. It was reported that IQGAP2 is SUMOylated, whereas SENP3 mediates the deSUMOylation of IQGAP2, and the host defense restores host protein synthesis and suppresses the expression of the HBV gene (Xi et al., 2019). NLRP3 (NACHT, LRR, and PYD domain-containing protein 3) is the best-studied NLR, which is stimulated by the pathogens and damage-associated molecular patterns (Hornung et al., 2008). NLRP3 inflammasome activation is crucial for host innate immune and inflammatory responses. SENP3 reverses the SUMOylation of NLRP3 to diminish ASC (apoptosis-associated speck-like protein with CARD domain) recruitment and speck formation, the activation of the NLRP3 inflammasome, and IL-1β cleavage and secretion (Shao et al., 2020). SENP3 mediates LPS (lipopolysaccharide)-induced coagulation activation by upregulating monocytes/macrophages TF (tissue factor) production via a JNK-dependent pathway (Chen et al., 2020). Taken together, these evidence shows that SENP3 regulates the inflammatory response via various signaling pathways.

## SENP3 Is a Key Regulator of Hepatic Lipid Metabolism in Non-Alcoholic Fatty Liver Disease

Non-alcoholic fatty liver disease (NAFLD) is characterized by excessive lipid accumulation and aberrant lipid metabolism in hepatocytes and has become an increasingly prevalent health problem worldwide (Rinella, 2015). Aberrant lipid metabolism plays a critical role in the progression of NAFLD and damages hepatocytes (Musso et al., 2009). A previous study showed that hepatic SENP3 expression level was increased after loading hepatocytes with free fatty acids (FFAs) *in vitro* and in an animal model and NAFLD patients *in vivo*. Silencing of the SENP3 gene was associated with amelioration of lipid accumulation, whereas overexpression of SENP3 enhanced lipid accumulation *in vitro*. Eleven unique genes, including apoe, a2m and tnfrsf11b, were regulated by SENP3 and closely associated with lipid metabolism. The protein levels of intrahepatic and circulating APOE, A2M and TNFRSF11B were strongly increased in NAFLD patients compared with those in controls. These studies identified the critical role of SENP3 in lipid accumulation and metabolism during the development of NAFLD through the regulation of downstream proteins (Liu Y. et al., 2016). It was concluded that the regulation of SENP3 might be a useful therapeutic strategy for the treatment of NAFLD through the transcriptional regulation of target genes.

## SENP3 Regulates SETD7 in Myopathy and Cachexia

Both myopathies and cachexia-associated muscle wasting showed similar features in the disordered structure of the sarcomere, leading to compromised sarcomeric function (Bossola et al., 2016). SENP3 specifically regulates the sarcomeric contractile myosin heavy-chain gene MyHC-II, affecting sarcomere assembly. Histone lysine methyltransferase SETD7 (SET domain-containing 7) can methylate lysine residues of proteins. SENP3 deSUMOylates and recruits SETD7 to MyHC-II and promotes the interaction of SETD7 with transcriptionally active RNA polymerase II. The expression level of SENP3 is degraded during cachexia, which is characterized by the tremendous loss of sarcomeric proteins, especially MyHC-II. In cachexia, SENP3 regulation of SETD7 is impaired, causing altered MyHC-II expression and disorganized sarcomeres (Nayak et al., 2019). These results revealed an important role of SENP3 in sarcomere assembly and cachexia.

## SENP3 Protects Against Osteoporosis

Bone homeostasis is important in body development, and osteoporosis is a bone metabolic disease with clinical features of disordered bone microstructure, systemic bone loss, and increased fragility (Schuit et al., 2004). Flightless-I homolog (FLII) is a direct interactor with SENP3, regulating the chromatin association of SENP3, thereby modulating MLL1/2 complex activity. Through the SENP3-mediated pathway, FLII regulates DLX3 gene expression, governing osteogenic differentiation in human mesenchymal stem cells (Nayak et al., 2017). Mice with the specific knockout of SENP3 in BMDMs (bone marrow-derived monocytes) exhibit much more severe bone loss due to osteoclast overactivation after ovariectomy. SENP3 knockout in BMDMs promotes the differentiation of osteoclasts. The deletion of SENP3 increases hyper-SUMOylation of IRF8 (interferon regulatory factor 8), and SUMOylated IRF8 increases the expression of NFATc1 (nuclear factor of activated T cell c1) and promotes osteoclastogenesis (Zhang et al., 2020). SENP3 mediated deSUMOylation of RbBP5, one of the SET1/MLL complexes. The deSUMOylation of RbBP5 is essential for HOX gene expression, as well as the developmental regulator DLX3. SENP3 deSUMOylation of SET1/MLL complexes-mediated DLX3 gene transcription plays a pivotal role during osteogenic differentiation in human stem cells (Nayak et al., 2014). These studies indicate that SENP3 is a key factor in protecting bones from osteoporosis.

## SENP3 Dynamically Regulates HIF1A and FIH1 Stability in Preeclampsia

Preeclampsia is a placental disorder that affects pregnancy and clinically arises in the third trimester with various maternal symptoms, including proteinuria, hypertension, and generalized edema (Sibai and Stella, 2009). It has been revealed that placental hypoxia and oxidative stress induce the genesis of preeclampsia, and the high expression level of HIF1A in preeclampsia is partially due to alteration of HIF1A hydroxylation (Rolfo et al., 2010). HIF1A stability is preserved in hypoxia, promoting nuclear accumulation, where it combines with HIF1A, thereby recognizing HRE (hypoxia-response elements) in the promoter of hypoxia-responsive targeting genes (Semenza and Wang, 1992; Maxwell et al., 1999). Several previous studies identified that HIF1A stability is also regulated by SUMOylation in hypoxic conditions (Cheng et al., 2007; Matic et al., 2008; Kunz et al., 2016). Thus, SENP3 and SUMOylation regulation is important in the cellular response to hypoxia by maintaining HIF1A homeostasis (Agbor and Taylor, 2008; Alahari et al., 2015). HIF1A SUMOylation peaked at 9–10 weeks, while SENP3-mediated deSUMOylation was highest at 10–12 weeks during placental development (Bhattacharjee et al., 2016). The factor inhibiting HIF1 (FIH1) was SUMOylated, and FIH1 SUMOylation directly impacted the transcriptional activity of HIF1A. There was enhanced FIH1 SUMOylation in early development, whereas SENP3-mediated deSUMOylation of FIH1 increased later in gestation (Sallais et al., 2017). In early-onset preeclampsia, both HIF1A and FIH1 deSUMOylation by SENP3 was significantly elevated in the human placenta, and this partly contributes to increased HIF1A activity and stability in physiological and pathological conditions (Bhattacharjee et al., 2016; Sallais et al., 2017).

## SENP3 Maintains Tight Junction Integrity and Cytoskeletal Architecture in Spermatogenesis

Spermatogenesis is a complicated process in which haploid spermatozoa develop from germ cells and thereby hold the heart of male fertility (Alves et al., 2013). The blood–testis barrier (BTB) is an important ultrastructure in the testis that erases the risk of infection of spermatogenic cells and the host immune response. The most distinctive and unique feature of the BTB is the presence of tightly packed actin filament bundles, which confer the unusual adhesive strength of the BTB. The actin filaments at the BTB will cyclically reorganize to adapt to tightly connected connection restructuring events, such as assembly and disassembly of various junction types, whereas actin depolymerization disrupts the tight junction structure and barrier function (Mok et al., 2013; Mruk and Cheng, 2015). SENP3 is expressed in spermatocytes and Sertoli cells in adult mouse testes. SENP3 regulates STAT3 transcriptional activity and affects the expression of genes involved in tight junctions, and knockdown of SENP3 disrupts the permeability barrier of tight junctions. Spermatogenesis requires actin-based cytoskeletal architecture, and this process is dynamically modified by SUMOylation (Alonso et al., 2015). On the other hand, SENP3 deficiency attenuates the organization of F-actin and inhibits orchestration of cytoskeletal architecture. SENP3 deficiency induced the dysfunction of regulators required for cytoskeleton architecture and tight junction integrity and ultimately destroyed the microenvironment for normal spermatogenesis (Wu et al., 2017). It was concluded that SENP3 plays a critical role in spermatogenesis via the maintenance of tight junctions and cytoskeletal architecture (Table 1).

## Concluding Remarks and Perspective

**FIGURE 2:**
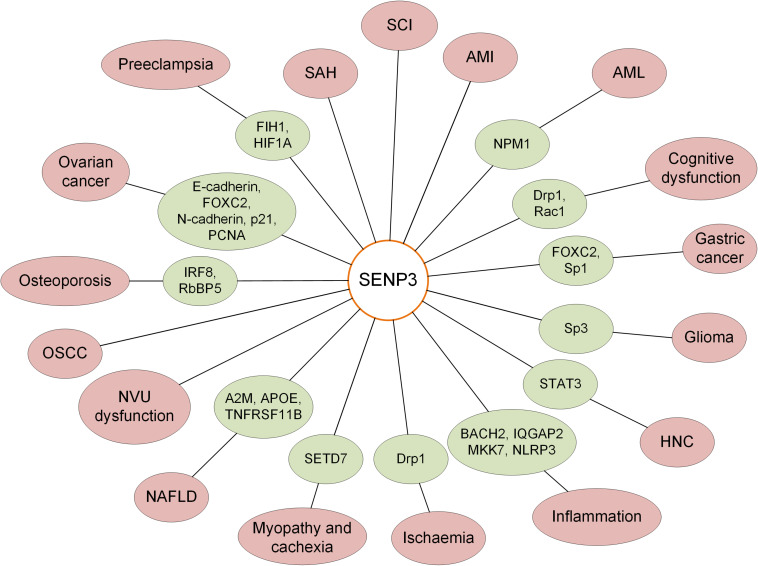
Roles of SENP3-mediated deSUMOylation of substrate proteins in various human diseases.

SENP3 and SUMO2/3 regulate the SUMOylation status of target proteins and play important roles in the maintenance of normal cellular functions. SENP3 regulates various pathways through variation of transcription activity or SUMOylation level of target genes or proteins. Previous studies have indicated that SENP3 mediates the balance of SUMOylation of numerous target proteins, which is closely correlated with the occurrence, development and progression of human diseases, including several kinds of tumors and neurological and cardiovascular diseases (Figure 2). Previous studies have shown that SENP3 levels vary in AMI, SAH and SCI; however, the specific roles of SENP3 in these diseases and the molecular mechanism require further investigation.

Increasing evidence has suggested that SENP accumulates in human diseases, and inhibition of SENP might be a potential approach for the clinical treatment of diseases (Kumar and Zhang, 2015). These previous studies indicate the potential significance of SENP3 as a diagnostic marker and make SENP3 an attractive drug target for the treatment of distinct human diseases. Therefore, SENP3-mediated deSUMOylation studies provide a theoretical basis for the pathogenesis and treatment of various diseases, and it may be promising to explore SENP3 inhibitors and their potential therapeutic roles.

## Author Contributions

XL, BZ, WL, and XC conducted the review. HW and YTQ conceived the project. XY, JH, YZ, SA, YYQ, ZX, and YS provided advice. HW and YTQ wrote the manuscript with editorial inputs from XL, BZ, WL, and XC. All authors contributed to the article and approved the submitted version.

## Conflict of Interest

The authors declare that the research was conducted in the absence of any commercial or financial relationships that could be construed as a potential conflict of interest.
